# Combined use of adapted Salmonella bacteriophage for isolation and elimination of large outbreak of nosocomial Salmonellosis

**DOI:** 10.1186/2047-2994-4-S1-O52

**Published:** 2015-06-16

**Authors:** V Akimkin

**Affiliations:** 1 State Budget Educational Institution of Higher Professional Education “I.M. Sechenov’s First Moscow State Medical University”, Moscow, Russian Federation; 2 Federal Budget Scientific Institution “Scientific Research Disinfectology Institute” of Federal Service for Surveillance on Consumer Rights Protection and Human Well-being, Moscow, Russian Federation

## Introduction

One of the major concepts of modern epidemiology and disinfectology is prevention and fighting with healthcare-associated infections (HAI).

## Objectives

Prophylaxis of HAI in Russia.

## Methods

Epidemiological, statistical.

## Results

It is one of the largest documented chronic outbreaks of Nosocomial Salmonellosis in the world which occurred (1994-1996) at the big multi-speciality hospital with more than 1,500 beds. The outbreak was active for approximately 3 years and more than 350 surgical and intensive care unit patients fell ill. At that point in time it was impossible to eliminate the outbreak by means of traditional antiseptics and antibiotics used for patients’ treatment.

For the first time ever in global practice adapted salmonella bacteriophage was used in a combined manner for treatment of NS patients (83 people), phagoprophylaxis of patients admitted to the in-patient hospitals and undergoing treatment there (more than 5,000 people), sanitation and phagoprophylaxis of staff (more than 2,500 people), and biological disinfection of more than 15,000 m^2^ of healthcare organization areas. Phagoprophylaxis schemes of patients found in NS effective disease area and undergoing treatment at in-patient hospital, and sanitation and phagoprophylaxis of stuff were developed and described by the author for the first time.

## Conclusion

The method of Salmonella Bacteriophage combined use was highly effective, harmless, non-toxic while treating patients with salmonellosis at surgical and intensive care units, it allowed performing effective disease prevention in risk groups and sanitation of staff, it was cost-effective and highly effective in decontamination of surfaces and objects in medical organization. Combined use of adapted salmonella bacteriophage allowed isolating and eliminating of long-existing effective disease area of Nosocomial Salmonellosis for 3 months from the time of bacteriophage’s application.

## Disclosure of interest

None declared.

